# Unraveling the genetic and molecular basis of heat stress in cotton

**DOI:** 10.3389/fgene.2024.1296622

**Published:** 2024-06-11

**Authors:** Aqsa Ijaz, Zunaira Anwar, Ahmad Ali, Allah Ditta, Muhammad Yousaf Shani, Sajjad Haidar, Boahua Wang, Liu Fang, Sana Muhy-Ud-Din Khan, Muhammad Kashif Riaz Khan

**Affiliations:** ^1^ Nuclear Institute for Agriculture and Biology College (NIAB-C), Pakistan Institute of Engineering and Applied Sciences (PIEAS), Islamabad, Pakistan; ^2^ National Key Laboratory of Crop Genetic Improvement, Huazhong Agricultural University, Wuhan, Hubei, China; ^3^ Nuclear Institute for Agriculture and Biology (NIAB), Faisalabad, Pakistan; ^4^ School of Life Sciences, Nantong University, Nantong, China; ^5^ State Key Laboratory of Cotton Biology, Institute of Cotton Research, Chinese Academy of Agricultural Science, Anyang, China

**Keywords:** high-temperature stress, cotton, epigenetic modification, transcriptomics, multiomics

## Abstract

Human activities and climate change have resulted in frequent and intense weather fluctuations, leading to diverse abiotic stresses on crops which hampers greatly their metabolic activities. Heat stress, a prevalent abiotic factor, significantly influences cotton plant biological activities resulting in reducing yield and production. We must deepen our understanding of how plants respond to heat stress across various dimensions, encompassing genes, RNAs, proteins, metabolites for effective cotton breeding. Multi-omics methods, primarily genomics, transcriptomics, proteomics, metabolomics, and phenomics, proves instrumental in studying cotton’s responses to abiotic stresses. Integrating genomics, transcriptomics, proteomics, and metabolomic is imperative for our better understanding regarding genetics and molecular basis of heat tolerance in cotton. The current review explores fundamental omics techniques, covering genomics, transcriptomics, proteomics, and metabolomics, to highlight the progress made in cotton omics research.

## 1 Introduction

Cotton is a perennial crop that belongs to the Malvaceae family but is cultivated as an annual crop. It is commercially cultivated in the USA, China, India, Brazil, Pakistan, Uzbekistan, and Australia (Egbuta et al., 2017). It is grown in more than 100 countries, covering an extensive land area of approximately 33 million hectares. As a major cash crop, it provides around 31% of the world’s fiber. Moreover, cotton contributes to a large extent in feed and food production around the world (Nabi et al., 2015; Mollaee et al., 2019). Additionally, cotton is also used as a source of edible oil and has some applications in the biopharmaceutical industry (Sheri et al., 2023a). It is usually classified into two types based on cultivation: wild and cultivated cotton (Han et al., 2022). There are 53 known species of cotton, four commercially cultivated, while the remaining 49 grow in natural habitats such as tropical and sub-tropical regions (Peng et al., 2021). *G. hirsutum*, *G. arboreum, G. herbaceum,* and *G. barbadense* are commonly cultivated and primarily grown for their fiber (Peng et al., 2021). However, these four species exhibited significant variations in fiber quality like fineness, maturity, length, and strength (Egbuta et al., 2017; Anwar et al., 2023).

Even though cotton production in the world has sustained at a constant rate during the last decade, there have still been significant variations in cotton crop production among various regions (Sheri et al., 2023a). The fluctuations in environmental conditions have reduced the crop productivity and sustainability because of alterations in their development patterns and ability to cope with severe conditions. The perpetual loss of agricultural land due to increased soil salinity, desertification, population expansion resulted in urbanization has exacerbated the consequences of climate change (Raza et al., 2019).

Various environmental factors like heat, drought, rainfall, humidity, and sunlight exposure impact the productivity of crops (Mukhtar et al., 2022; Shokat et al., 2023; Victoria et al., 2023; Hafeez et al., 2024). However, among these ecological factors, high temperature stress (HTS) greatly affects the growth and development of several crops, including cotton (Yousaf et al., 2023). Singh et al. (2007) reported that the lint yield of cotton could decrease by up to 110 kg/ha if the temperature increases by 1 °C above the optimal growth range (Singh et al., 2007). The flower stage of cotton is significantly sensitive to HTS, leading to significant flower abscission, stunted plant growth, and reduction in boll weight and number of bolls (Zafar et al., 2022). The temperature ranges from 32°C to 40°C adversely affects root development and stomatal conductance. A temperature above 29°C could also reduce boll weight (Lokhande and Reddy, 2014). The temperature above 37°C significantly impacts the seed germination rate, leading to elongation in both pollen germination and pollen tube (Burke et al., 2004). Salman et al. (2019a) reported that a reduction in cotton yield could be associated with a decrease in net photosynthetic rate and sympodial branches while bolls and flowers start to shed under HTS (Salman et al., 2019a).

Alongside the reduction in cotton yield, HTS also reduces fiber fineness and uniformity and decreases lint quality by increasing short fibers (Khan et al., 2022b). HTS can detrimentally influence all stages of plant growth and their development, participating in both direct and indirect damage (Ullah et al., 2016). However, susceptibility to HTS differs amidst phenological stages and varies among species and genotypes of the same species. HTS caused various types of injuries like leaves and twigs, necrosis and chlorosis in branches, leaves, and stems, premature senescence and abscission of leaves, flower, and fruit, inhibition of root and shoot growth, alteration in leaf angle, changes in transpiration rates, seed shrinkage and alterations in phospholipid extracellular matrix components. Consequently, these injuries result in lower plant productivity (Vollenweider and Günthardt-Goerg, 2005). Even when plants are subjected to HTS, they usually close their stomata to decrease water loss by enhancing the tracheids and stomata frequency and expanding vessels of the xylem, which helps the plant cope with HTS (Abro et al., 2023). The signal transduction system is important in trigging the self-regulated and hormone-meditated mechanism under HTS in plants (QAMER et al., 2021).

Reactive oxygen species act as important signaling molecules and perform diverse functions by responding rapidly to environmental factors, but when accumulated in excess quantity, ROS becomes toxic for plants (Zahra et al., 2021). Under HTS, cell biochemistry is negatively influenced by the production of ROS, which alters the function of mitochondria and subsequently instigates oxidative damage due to lipid peroxidation (Mansoor and Naqvi, 2013; Zahra et al., 2023). Various studies reported enhanced lipid peroxidation under HTS conditions (Wu et al., 2010). HTS induces the production of ROS like OH^−^, H_2_O_2_, and O_2_^−^, leading to oxidative stress (Yin et al., 2008). The rubisco enzyme is responsible for the increased production of H_2_O_2_ via its oxygenase activity under HTS (Kim and Portis, 2004). ROS instigates autocatalytic peroxidation of lipids in cell membranes and pigments, causing membrane damage that influences its function and permeability (Xu et al., 2006). For example, when cotton plants experienced HTS during the reproductive stage, a higher level of ROS coupled with elevated level of lipid peroxidation was found, which is damaging for all cell organelles. Moreover, cotton plants encounter difficulty to eliminate ROS under HTS (Zhang et al., 2016; Zafar et al., 2023).

Plants have developed a two-pronged defense mechanism to protect themselves from oxidative stress. The defense system comes into action by activating various enzymes like ascorbate peroxidase and superoxide dismutase (SOD) and non-enzymatic elements like phenolic acids and flavonoids. The non-enzymatic and enzymatic systems work harmoniously to eliminate free radicals or interrupt their reactions by breaking them down (Poudel and Poudel, 2020). Plant defense mechanisms encompass various enzymatic elements, including ascorbate peroxidase (APX), ascorbate (ASC), glutathione (GSH), and superoxide dismutase (SOD). Antioxidants defend molecules from oxidation, neutralize free radicals, and transform them into less reactive forms. Thus, antioxidants play an important role in achieving a balance between ROS production and ensuring the proper functioning of cells. HTS influences antibodies in cell membranes, which makes them sensitive to denaturation under oxidative stress. Heat can affect antibodies in cell membranes, rendering them susceptible to denaturation under oxidative stress. Therefore, recognizing non-enzymatic and enzymatic systems could be associated with stress and used as an indicator of stress tolerance. Proline, a non-enzymatic antioxidant, acts as an osmoprotectant and plays a significant role in various stress signaling pathways, helping the plant endure harsh conditions. The higher level of proline accumulation under stress is crucial, as proline efficiently scavenges reactive oxygen species (ROS) from the cells (Alagoz et al., 2023). This protects the cells from damage caused by reactive oxygen species (ROS) while sustaining their regular biological functions (Alagoz et al., 2023).

Molecular signaling is triggered when newly synthesized proteins are released from the ribosome, and substrate binding domains of Heat Shock Protein (HSP70) help to identify and bind to hydrophobic amino acid residue sequence (Faust et al., 2020). The heat shock protein gene is important in plants, as it helps the plant’s cellular machinery defend against various abiotic stresses such as heat. It also performs an additional role by interacting with extended sections of protein peptides and with proteins that are folding. The interaction of *HSP70* with other proteins prevents them from aggregating together, facilitates changes in their folding to reach their final shape, and controls how the proteins perform (Usman et al., 2017; Waudby et al., 2019). Many *HSPs* have been extracted from several organelles like plastids, the cytosol, and the endoplasmic reticulum of many different plant species under various abiotic stresses (Roy et al., 2019). These *HSPs* act as chaperones, proving their role in tolerance against heat and other stresses (Tang et al., 2016).

There is a dire need to understand the genetic and molecular basis of the cotton plant under HTS and how it responds to high temperatures. To sustain crop productivity in the era of climate change, private companies, breeders and publicly funded institutes must incorporate faster and more productive strategies to fast-track heat-tolerant cotton development. Traditional breeding involves crossing plants with desired traits to develop a cultivar. Breeders perpetually conduct crosses over multiple generations, involving crossing and then selection, eventually selecting plants that have the best blend of desired traits (Derbyshire et al., 2022; Ahmed et al., 2023). This could be achieved by improving traditional breeding methods through the incorporation of genomic data or the precise manipulation of genetic determinants and metabolic pathways using advanced techniques such as genome editing. Over the last decade, breeders have utilized genomic methods to improve breeding and expedite cultivar development (Ahmar et al., 2020). For instance, some crucial phenotypes are commonly seen late in the breeding cycle. It has become feasible to recognize plants much earlier by using predictive models based on marker data, potentially eliminating the need for multiple generations and several years of breeding (Fu and Yuna, 2022).

Henceforth, this manuscript aims to explore the genetic basis of cotton and the application of advanced omics techniques such as transcriptomics, genomics, proteomics, and metabolomics in studying the response of cotton species to HTS. The purpose is to comprehend how genotype, environment, and epigenetics together contribute to the development of phenotypes that are tolerant or resistant to HTS.

## 2 Genetics of HTS tolerance in cotton

The ancestors of cotton (*Gossypium* genus) originated and grew in the semi-arid (dry-hot) habitat (Baytar et al., 2022); thus, cotton is a heat-loving plant and grows well in the tropical and subtropical regions. However, the changing climate and the increasing global temperature threaten the cotton plant’s survival and limit its sustainable productivity (Iqbal et al., 2017; Rani et al., 2022). HTS occurs at various stages of the lifecycle, potentially limiting cotton yield and quality. HTS affects cotton plants at multiple stages but is more lethal during the flowering stage. High temperature is injurious to anther and pollen, which causes male sterility and impaired pollen viability, eventually reducing boll setting and final yield (Khan et al., 2020) Exposure of cotton to high temperature for longer duration causing leaf wilting and boll shedding, and reduced photosynthesis (Yun-Ying et al., 2008; Iqbal et al., 2017). Therefore, it is important to focus on identifying genetic resources with important QTLs and developing new germplasm that carry the QTLs, which can cope with HTS (Salman et al., 2019b). High temperature or heat tolerance (HTT) of the cotton cultivars can guarantee optimum yield during stress conditions. HTT is a complex trait controlled by multiple QTLs across the genome (Rani et al., 2022). HTT is conferred by several other associated traits, i.e., stomatal conductance, canopy temperature, transpiration, and serval other biochemical traits. All of these traits are of a complex quantitative nature and regulated by several epistatic QTLs. To gain insight into the genetic and molecular mechanism of HTT, it is essential to comprehend the genetic and molecular mechanisms of the related traits. Unravelling the genetic architecture of HTT will not only be helpful for basic research, but also hold importance in breeding thermos-tolerant cotton cultivars for sustainable production under the changing climate.

The recent advances in plant biotechnology have experienced great advances, particularly in the fields of genomics, genome sequencing, and marker technology. Introducing these technologies has provided great ease to plant scientists (Ma et al., 2021a; Baytar et al., 2022). QTL mapping is widely employed in plant breeding, proving to be an effective tool for unraveling the genetic basis and architecture of complex traits and facilitating marker-assisted breeding (Holland, 2007), locating regions on the genomes affecting the phenotype of an organism and map-based cloning. With the development in the field of genomics and the advancement of molecular marker technology, the next-generation sequencing (NGS) strategies also have fast-tracked the QTL analysis (Kumar et al., 2017). QTL mapping serves as a tool to identify the genes that influence the traits, locate the gene position on the genome, step towards map-based cloning, marker-assisted selection (MAS) (Khan, 2015; Khan et al., 2022b). Linkage mapping, association mapping and bulk segregant analysis (BSA) have been used in routine to identify the QTLs and gene controlling quantitative traits in various crops. These approaches have also been utilized to identify QTLs regulation tolerance to abiotic stresses.

In cotton, QTL mapping has been extensively applied to uncover the genetic mechanism of quality and yield traits (Kushanov et al., 2021). Various studies have reported several Single Nucleotide Polymorphisms (SNPs), Quantitative Trait Loci (QTL), and QTL clusters associated with fiber quality traits (Fang et al., 2014; Islam et al., 2016; Liu et al., 2020). Similarly, the genetic basis of biotic and abiotic stresses have been focused in detail (Dabbert and Gore, 2014). Progresses have been made to identify QTL regulating resistance against wilt infection (Li et al., 2017; Zhang et al., 2019; Abdelraheem et al., 2019; Abdelraheem et al., 2020; Abdelraheem et al., 2021), Li et al. (2020) have reported QTL regulation tolerance under drought and salt stress. However, the genetics basis of HTS response and HTT in cotton remains elusive, and very few loci have been identified. The HTT associated loci have been summarized in Table 1. The majority of these loci have been identified in combined stress conditions, i.e., drought and high aerial temperature, or dry arid conditions (Ulloa et al., 2000; Saranga et al., 2004), due to fact that both stresses often happen at same time. Ulloa et al. (2000) identified two putative loci for stomatal conductance in the F3 families developed from the cross between NM24016 and TM1 (Ulloa et al., 2000). One of these loci, G3800 showed over-dominance effect on the expression of stomatal conductance in heterozygous state. Interspecific hybridization is used as an effective tool for gene/alleles introgression from the wild relative into cultivated crop species. Saranga et al. (2004) developed an F_2_ population (n = 406) and subsequent F_3_ families via interspecific cross between *G. hirsutum* (cv. Sivon) X *G. barbadense* (cv. F-177) (Saranga et al., 2004). Interestingly, 33 loci were identified in the F_2_ population for physiological traits, including canopy temperature and chlorophyll contents under stress conditions. Similarly, Debbert et al. (2014) identified several loci regulating fiber quality and agronomic characteristics under HTS conditions in two RIL populations (Dabbert, 2014). In a RIL population obtained from the cross between TM-1 and M24016, Pauli et al. (2016) pinpointed numerous loci associated with canopy temperature and other related traits under hot arid conditions (Pauli et al., 2016). Canopy temperature related loci were identified on Chromosome A01, A08, A09, A13, D10, and D12 Interestingly, some of these loci overlapped with other loci regulating agronomic traits. GWAS analysis coupled with transcriptome wide association analysis (TWAS), Ma et al. (2021a) identified some loci that negatively regulated affected the pollen viability and caused male sterility (Ma et al., 2021a). Further, the TWAS analysis confirmed that *GhHRK1* (*Ghir_A01G006180*) negatively regulates anther development under HTS. Rani et al. (2022) developed an F2 population from MNH-886 (heat tolerant) and heat sanative (MNH-814) parents. This F_2_ population carried 17 QTLs for agronomic and physiologic trait under HTS (Rani et al., 2022). Overall, it can be summarized, that the genetics of HTT is not well studied compared to other stresses in cotton, as well as other crops. Due to the increasing areal temperature and prevalent arid dry conditions during cotton growth, it is of utmost importance to analyze the genetics of HTT and identify the important genes that confer HTT.

**TABLE 1:** Quantitative trait loci (QTLs) identified for HTS tolerance in cotton.

Loci	Chromosome	Population	Stage	Associated trait	Gene	Approach	Reference
*S3, G3800*	*LG17, LG20*	118^a^ F2.3 progenies (s NM24016/TM1)	Flowering stage	Stomatal conductance, lint yield		Linkage mapping (CIM)	Pauli et al. (2016)
*pAR3-32a, pAR402b, pAR606, pAR248, pGH232a*	*Chr06, LGA02, LGA05, LGD03*	406 F2 (G. hirsutum cv. Siv’on × G. barbadense cv. F-177)	Boll opening stage	Canopy temperature, osmotic potential, chlorophyll contents	*--*	Linkage mapping	SARANGA et al. (2004)
*--*	*--*	RILs: MAB1 (117), MAB2 (113)	--	Agronomic and fiber traits	*--*	Linkage mapping (CIM)	Dabbert (2014)
*--*	*A01, A08, A09, A13, D10, and D12*	95 RILs (TM-1 x M24016)	--	Canopy traits, LAI	*Gh_A13G0355*	Linkage Mapping (ICIM)	Pauli et al. (2016)
*A01_9098282, D01_8274957, D05_23761911*	*A01, D01, D05*	218 natural inbred accessions	Flowering stage	Pollen viability	*Ghir_A01G006180, Ghir_D01G006520, Ghir_D01G006540*	GWAS, TWAS	Ma et al. (2021a)
***--***	*2, 3, 5, 6, 15, 16, 18, 19, 23, and 26*	94 F2 progenies (*MNH-886 x MNH-814*)	---	Agronomic and physiologic traits	*--*	Linkage mapping (CIM)	Rani et al. (2022)

^a^ Number of accessions, CIM: Composite Interval Mapping.

## 3 Transcriptome alteration during HTS in cotton

Transcriptome analysis reveals gene expression differences, giving insights into the functions of genes. The advancements in RNA profiling technologies, such as microarrays and RNA-seq, have made transcriptome analysis easier (Duque et al., 2013). High-throughput sequencing has enabled larger-scale transcriptome analysis (Pandit et al., 2018). Larger transcriptome datasets obtained from various individuals or conditions provide valuable data about the regulatory responses of plants to their environment. Current studies have used a transcriptomic approach to understand abiotic stress pathways in multiple crops. The transcriptomics tool helps to identify stress-responsive genes to compare stress tolerance and control gene expression (Agarwal et al., 2014). Until now, this technique has unveiled various stress responsive genes and their expression pattern under environmental stresses in numerous crops like wheat, maize, barley, sorghum, rice, cotton and soybean (Baillo et al., 2019; Javaid et al., 2022; Kopecká et al., 2023).

Several studies have deciphered the molecular responses to heat stress (HTS), which lead to indehiscence and pollen sterility, resulting in lower yield in cotton plants. Genes associated with pollen and anthers play important roles in male reproduction and the response to HTS. Peng et al. (2016) also found 4,698 genes differentially expressed after 4–8h under HTS when comparing heat-tolerant (NH) and heat-sensitive (E7) cotton genotypes. Many heat-induced differentially expressed genes (DEGs) showed higher expression in NH or were unique to NH, encoding protein kinases, transcription factors, and heat shock proteins important for thermo-tolerance. Key possible thermo-tolerance regulators included two heat shock transcription factors (*AtHsfA3*, *AtHsfC1* homologs) and four *AP2/EREBP* genes (*AtERF20*, *AtERF026*, *AtERF053, AtERF113* homologs) (Peng et al., 2016). Zhang et al. (2022) performed comparative transcriptomics on anther and pollen from high-temperature sensitive lines under both controlled and high temperature in cotton and identified 1,066 genes specific to anthers and 1,111 genes specific to pollen. Under heat stress, 833 genes were differentially expressed in pollen when analyzed and compared. Ten pollen-specific genes responsive to heat stress were found (Zhang et al., 2022). Ma et al. (2021b) discovered that the heat-susceptible gene *GhHRK1* exhibits a negative association with HTS in various crops like cotton (Ma et al., 2021a). Interestingly, its Arabidopsis mutant imparts heat tolerance, suggesting a significant role in heat resistance. Genes involved in cytokinin, ABA, and brassinosteroid signaling like *CRE1, ABF*, and *CYCD* may regulate cotton’s heat response and sustain growth (Prerostova et al., 2020; Zhao et al., 2020). Similarly, Liang et al. (2021b) identified long non-coding RNAs (lncRNAs) targeting essential candidate genes associated with high-temperature tolerance in cotton. These candidate genes encompass chlorophyll a-b binding proteins, ribosomal proteins, and heat shock proteins. Examination of the expression profiles of the anticipated lncRNAs revealed higher expression levels in the heat-tolerant cultivar compared to the heat-sensitive cultivar under conditions of HTS. The roles of circular RNAs (circRNAs) in the growth, development, and stress response of cotton were investigated (Liang et al., 2021a; Wang et al., 2024). They performed bioinformatics analysis on RNA sequencing data from pollen grains of two near-isogenic cotton lines, NH and SH which differ in fertility stability under high temperature (HT) stress. In total, 967 circRNAs were identified in the cotton pollen grains, of which 250 were differentially expressed under high temperature stress. A detailed list of genes identified by using transcriptome in cotton under heat has been given in Table 2.

**TABLE 2:** Putative genes identified via transcriptome analysis in cotton under HTS.

Genes/TFs	Tissue/Stage	Heat treatment	Pathway/Function	Reference
*QRT3* and *CYP703A2*	Anther	38°C–40°C day, 28°C–31°C night (7 days)	abnormal pollen wall and male sterility	Li et al. (2023b)
*GhACO2*	Flower	10 days of high temperature stress	fatty acid and jasmonic acid metabolic pathways involve in male sterility	Khan et al. (2020)
*Ghir_D12G012350*	Anther	35°C–39°C day, 29°C–31°C night	involved in the process of pollen development while responding to high temperature stress	Zhang et al. (2022)
*CKI genes*	Anther	35°C–39°C day, 29°C–31°C night	involved in anther development	Li et al. (2021b)
*SPLs*	Flower	39°C ± 2°C day, 29°C ± 2°C night (7 days)	exogenous Indole-3-acetic acid (IAA) leads to a stronger male sterility	Ding et al. (2017)
*amylase genes*	Anther	39°C–41°C day, 29°C–31°C night	auxin biosynthesis and signaling pathways	Ma et al. (2018)
*ARF10* and *ARF17*	Flower	39°C ± 2°C day, 29°C ± 2°C night (7 days)	exogenous Indole-3-acetic acid (IAA) leads to a stronger male sterility	Ding et al. (2017)
*GhHRK1*	Flower	maximum temperature was greater than 37°C for 3 days	involve in male sterility in cotton	Ma et al. (2021b)
*GhCKI* and *PIFs*	Anther	35°C–39°C day, 29°C–31°C night	increase in indole-3-acetic acid level in late-stage anthers	Min et al. (2014)
*AtHsfA3, AtHsfC1*	true leaves	45°C for 8 h	key regulators of thermo-tolerance in cotton	Peng et al. (2016)
*AtERF20, AtERF026, AtERF053,* and *AtERF113*	true leaves	45°C for 8 h	key regulators of thermo-tolerance in cotton	Peng et al. (2016)
*GhHSFA2* and *GhHSFA1a*	true leaves	42°C	positively modulated thermo-tolerance in cotton	He et al. (2022)
*GhHXK1* and *GhERF1A*	true leaves	42°C	negatively modulated thermo-tolerance in cotton	He et al. (2022)
*HSFs*	true leaves	40°C for 4, 8, and 12 h	insights into the potential heat-tolerant mechanism in cotton	Liang et al. (2021a)
*GhCKI*	Anther	35°C–39°C day, 29°C–31°C night	GhMYB66–GhMYB4–GhCKI regulatory pathway	Li et al. (2022)

## 4 Proteomic profiling under HTS in cotton

Proteomics plays an imperative role in investigating of biochemical processes and plant responses to various abiotic stresses, such as HTS (Timperio et al., 2008; Katam et al., 2020). Plant stress proteomics can be utilized to elucidate potential key regulatory genes for genetically improving cotton’s resistance to extreme heat conditions (Ahmad et al., 2016). Plants respond differently against multiple harsh environmental extremes and assist in regulating cell homeostasis. Various proteins (HSPs and HSFs) have been recognized as essential for developing stress resilience in cotton (Manna et al., 2021).

Crop plants like cotton actuate several mechanisms in response to HTS, including physio-biochemical and molecular responses (Yadav et al., 2020). According to their molecular weight, these HSP*s* are categorized into various categories, including small HSPs, HSP60, HSP70, HSP90, and HSP100 (Khan and Shahwar, 2020). In cotton, HSPs primarily act as molecular chaperones and are in charge of the folding of proteins, transport, accumulation, and destruction (Usman et al., 2014). For instance, Hsp90 is involved in the translocation of signaling proteins, including actin, calmodulin, and kinases. Despite this, the interaction between HSPs and HTS is strongly correlated, and the development of a transformed cotton plant with a desired gene of interest encodes the synthesis of *HSP101* protein perceived in *Arabidopsis thaliana*. So, the transgenic cotton plant enhances pollen germination rate and pollen tube elongation under HTS conditions (Nadeem et al., 2018). For example, *Hsp70* assists in preventing protein refolding from accumulating and protecting cells from damaging effects caused by endoplasmic reticulum (ER) stress, while *Hsp90* plays a crucial role in the translocation of signaling proteins (kinases, calmodulin, actin, etc.) (Nadeem et al., 2018). Likewise, by breaking down misfolded proteins, tiny *HSPs* decrease the build-up of irreversibly unfolded proteins and support plants for adaptation against HTS (Hoter et al., 2018). A transgenic cotton plant with a gene encoding the *Arabidopsis thaliana HSP101* protein was formed, providing solid evidence for the link between HT and *HSP*. Under high temperatures, transformed cotton plants generate pollen that has a higher germination rate and longer elongation pollen tubes. Additionally, binding proteins (BiP) directly associated with the classical *HSP-70* family, bind with newly prepared proteins and keep them in folded form. BiPs are involved in protein translocation and activation of unfolded proteins response directly linked with the basic leucine zipper 28 (Bzip28) stress sensor of the endoplasmic reticulum (ER). The transfer of heat shock protein related gene (*AsHSP70*) in *G. hirsutum* was conducted via Agrobacterium, and the expression of transgenic cotton species under high-temperature constraints at the molecular, physiological, and biochemical levels only 1.9% transformation efficiency was found using genetic transformation (Batcho et al., 2021). Transgenic plant genomic DNA contained an 1800 bp fragment of *AsHSP70*, which was amplified by PCR. In transgenic cotton plants, polyamine oxidase increased the relative expression of *AsHSP70* from 1.02 to 9.58 as the length of heat stress increased. In the transgenic plant, *AsHSP70* expression was comparatively higher in the leaves than in the root and stem during the combined heat and drought stress (Anaraki et al., 2018).

HTS and severe drought occurrence pose constrains in the lint quality and quantity improvement. A complete characterization and harnessing Hsf gene family in *G. hirsutum* is essential for understanding the roles of several *Hsfs* across the genomic level (Fan et al., 2021), particularly in HTT. In cotton (*G. hirsutum*), heat shock transcription factor (*Hsf*) genes were cloned and identified using genome-wide analysis and EST assembly (*GhHsf*). Cloning, identification, and classification of forty (40) *GhHsf* genes in three primary classes (A, B, and C) based on their domains properties will assist in identifying and better understanding of the key heat shock transcription factors involved in developing heat resilience in crop plants (Wang et al., 2014; Chang et al., 2017; Liang et al., 2021b). The expression pattern of *GhHsf* related transcripts in the majority of tissues in cotton crop, including morphological characters such as leaves, stems, roots, and fibers synthesis way. Results of quantitative real-time PCR (qRT-PCR) aid in determine the elevated expressiveness level in developing ovules of cotton under heat stress scenario. A comparative investigation of the expression patterns between fiber less and wild-type mutants which showed that *Hsfs* are involved in the formation of cotton fiber. The upland cotton genome has around 80 *Hsf* genes owing to duplication of genome, and the D-sub genome alone has 40 Hsf-related genes. The heat shock-induced expressions in several tissues demonstrated the importance of *GhHsfs* for both heat stress and fiber formation (Wang et al., 2014; Huang et al., 2022).

Due to major abiotic extremes such as drought and heat stressors, it was observed that the new transcription factors, i.e., Cys-2/His-2-type zinc finger (C2H2-ZF), regulate stomatal aperture (Han et al., 2020). WRKY TFs play a crucial role in wheat response to abiotic stress. In cotton, non-coding RNAs, phytohormones, and smaller-sized peptide formation are thought to be important elements that carry out gene functions in response to abiotic stress conditions (Patra et al., 2023). Several TFs, such as dehydration-responsive element/C-repeat (DRE/CRT) and DRE/binding protein 2 (DREB2), regulate a variety of phytohormone-independent toward harsh environmental stresses in tetraploid cotton (Li et al., 2020; Sadau et al., 2024). According to transcriptome analyses conducted on cotton under heat stress, certain genes are differentially expressed (DE) to combat stress scenarios (Ma et al., 2021b). Distinct patterns of *OsMADS* gene expression were discovered in cotton crops that were still developing in response to drought stress (Premkumar, 2023). These transcriptome sequencing investigations may be helpful for functional evaluations as their expression pattern is differential during crop development and growth stress response. Thus, these studies highlight the significance of transcriptomics for proper crop development and response against various environmental constrains. The comprehensive analysis of the proteome under heat stress is presented in Table 3.

**TABLE 3:** Various proteins identified via proteomics analysis in cotton under heat stress.

Protein	Tissue/Stage	Heat treatment	Technique	Effects	Reference
HSP70-17 and BiP5	anther	35°C–39°C day, 29°C–31°C night	MALDI-TOF/TOF technique	involved in the reduction of the ROS effect on pollen grains under high-stress	Khan et al. (2022a)
Rab proteins	pollen at the anthesis stage	36°C or 40°C (5 days)	nanoLC-MS/MS technique	involved in the increase of pollen thermo-tolerance	Masoomi-Aladizgeh et al. (2022)
HSPs	leaves	45°C	RT-qPCR	-	Ali et al. (2022)
transport protein Sec61	leaves	40°C	RT-qPCR	involve in the degradation of misfolded proteins	Liang et al. (2021a)
Hsp70s and cytoskeletal proteins	squares	40°C day, 30°C night, (5 days)	Mass Spectrometry	involve in the activation of key pathways to control protein folding	Masoomi-Aladizgeh et al. (2021)
AsHSP70	leaves	45°C	Transformation method	confirmed the higher mRNA expression in different plant tissues under heat stress	Batcho et al. (2021)
GhHRP	leaves	45°C for 4 h	-	Act as heat-responsive signaling component	Abdullah et al. (2023)
Histone 3	anther	38°C–40°C day, 28°C–31°C night (7 days)	ChIP-seq	maintain male fertility in cotton under heat stress	Li et al. (2023a)
Lysine 4	anther	38°C–40°C day, 28°C–31°C night (7 days)	ChIP-seq	maintain male fertility in cotton under heat stress	Li et al. (2023a)
HSP101, HSP3	-	45°C	Morpho-physiological and molecular analysis	lead to higher photosynthesis, cell membrane stability	Saleem et al. (2021)
Rubisco	leaves	37°C	2-DE technique	could maintain photosynthesis at higher temperatures	Smith et al. (2023)
HSP3	leaves	42°C	Western blotting and immunostaining assay	involve in enhancing thermo-tolerance in cotton	He et al. (2022)

## 5 Metabolomics profiling under HTS

Plants, being sessile organisms, are unable to avoid environmental conditions. Consequently, they are constantly subjected to abiotic stresses, which can have adverse effects on their growth and development, ultimately impacting productivity and crop yields-an issue that has been previously emphasized. Thus, there is need of day to completely understand the biochemical nature of plant responses to abiotic stresses. Hence, we would comprehend and predict the metabolic activity of plants under harsh conditions (Hasanuzzaman et al., 2013; Zhang et al., 2023). In the light of recent advancements in ‘omics’ technologies, metabolomics is emerging as a promising tool of plant biology. It involves the synthesis of information from gene expression, protein interactions, and pathway regulations (Weckwerth, 2003). Metabolomics primarily focuses on widespread quantitative and qualitative investigation of all small molecules of biological organisms. It involves the thorough analytical examination of low molecular-weight compounds known as metabolites found within biological specimens, all within specified conditions (Oh et al., 2023; Shokat et al., 2023). Metabolomic analysis can target either a predefined set of compounds or encompass the entire metabolome. NMR (Nuclear Magnetic Resonance) and MS (Mass Spectrometry) are the predominant techniques utilized for generating comprehensive metabolomic profiles (Misra and van der Hooft, 2016).

Plants contain a vast array of metabolites, which can be categorized into primary and secondary types (Arif et al., 2022). Primary metabolites are crucial for synthesizing key macromolecules such as carbohydrates, lipids, and proteins, and they play essential roles in fundamental biological processes like the tricarboxylic acid cycle (TCA), glycolysis, and fatty acid biosynthesis (Razzaq et al., 2019). These metabolites serve as the final products of cellular activities, and changes in their concentrations can serve as indicators of the overall condition of the entire plant. The primary objective of investigating metabolic alterations in plants is to uncover metabolic characteristics, often referred to as metabolic biomarkers, which help restore cellular balance and normal metabolic functioning in stressed plants. These biomarkers play a crucial role in facilitating stress response and tolerance (Katam et al., 2022).

The plant metabolome, comprising all low molecular weight metabolites synthesized by plant cells under specific conditions at a given time, influences processes at the macro-molecular level. These processes can be studied using various analytical techniques (Allwood et al., 2011). Metabolomics analysis has transformed our understanding of phytochemicals by enabling comprehensive profiling and characterization of both primary and specialized secondary metabolites in plants across different environmental conditions. This is especially notable in examining responses to both abiotic and biotic stresses, as well as resistance and tolerance mechanisms (Salam et al., 2023). Metabolomics has proven valuable in various crops such as soybean, rice, corn, barley, tomato, maize, and wheat, particularly for studying stress conditions (Razzaq et al., 2019). Nonetheless, its targeted use in assessing heat stress in cotton remains untapped.

In Arabidopsis, metabolic profiling has unveiled the temporal dynamics linked with heat stress (Kaplan et al., 2004). Likewise, metabolomic investigations in grapes have explored drought and salinity stresses, revealing changes in metabolic pathways concerning gluconeogenesis, energy metabolism, and nitrogen assimilation (Cramer et al., 2007; Rabara et al., 2017 found that gradual dehydration led to changes in the root metabolome, resulting in the accumulation of 4-hydroxy-2-oxoglutaric acid and coumestrol in tobacco and soybean roots (Rabara et al., 2017). In 2017, Aayudh Das noted that essential metabolites, including carbohydrates, amino acids, lipids, cofactors, nucleotides, peptides, and secondary metabolites, exhibited varying accumulation patterns under heat and drought stress conditions in soybean leaves. Drought and heat stress were observed to impact a range of metabolites involved in essential cellular processes, including glycolysis, the tricarboxylic acid (TCA) cycle, the pentose phosphate pathway, and starch biosynthesis. These processes regulate carbohydrate metabolism, amino acid metabolism, peptide metabolism, as well as purine and pyrimidine biosynthesis. During heat stress, wheat demonstrated elevated levels of sucrose and G1p, while soybean exhibited increased concentrations of 1,3-dihydroxyacetone, ribose, glycolate, and other metabolites (Das et al., 2017). These discussions imply that metabolomics serves as a valuable tool for comprehending plant responses to stress conditions. Metabolomics offers valuable insights into how plants respond to particular stress conditions by altering their internal biochemistry, thereby mitigating harmful effects (Villate et al., 2021).

Currently, there is limited knowledge available regarding the cotton metabolome during stress conditions, particularly in the context of heat stress. Metabolomics combined with HTP-phenomics will significantly enhance our understanding of how cotton responds to heat stress. Integrating metabolomics with genomics and transcriptomics will assist in identifying potential genes and pathways responsible for generating responses.

## 6 Epigenetic responses during HTS in cotton

The rise in global temperatures has elevated extreme high-temperature events, leading to an increase in abiotic stress on terrestrial plants (Zhao et al., 2020; Verma et al., 2022). Plants, being sessile organisms and anchored to soil, are unable to evade the environmental extremes (HTS in particular). Therefore, plants activate complex physiological and molecular or gene networks in response to these stresses. However, the complete understanding of these responses still needs to be understood. Beside the physiological and molecular network, epigenetic modifications and regulations are also considered important in stress condition. Recently, it has been observed that epigenetic regulations play efficient role in the plant’s survival under various abiotic stresses (Verma et al., 2022). Epigenetic modifications encompass changes in the chromatin structure of stressed genes through various chemical modifications during both transcriptional and post-transcriptional events (Kim et al., 2015; Lämke and Bäurle, 2017). Molecular mechanisms of epigenetic regulation mainly consist of DNA methylation, chromatin/histone modifications and small non-coding RNAs, etc (Grewal and Rice, 2004; Huang et al., 2016). These changes aim to alter the expression of responsive genes under stress conditions. These alterations also create stress memory that can be passed on to the succeeding generation (Friedrich et al., 2019). Hence, the understanding of epigenetic responses and its consequences become more important in crop species particularly in cotton under the increasing Arial temperature and HTS.

Several plants species have been reported to exhibit epigenetic modification in response to abiotic stresses. Compared to other stresses (drought and salinity), less information is yet available about the epigenetic regulations and modification under HTS. The Arabidopsis *HDA9* interacts with the PWR protein, leading to enhanced *H3K9* deacetylation of *PIF4* and *YUC8* (Tasset et al., 2018; Van Der Woude et al., 2019). Similarly, under normal temperatures, *HDA15* suppresses warm-temperature-related genes such as *ATHB2*, *XTR7, YUCCA8*, *IAA19* and *IAA29*, while activating these genes under HTS (Shen et al., 2019). An increased DNA methylation have been reported in *B. napus* under HTS (Gao et al., 2014).

**FIGURE 1:**
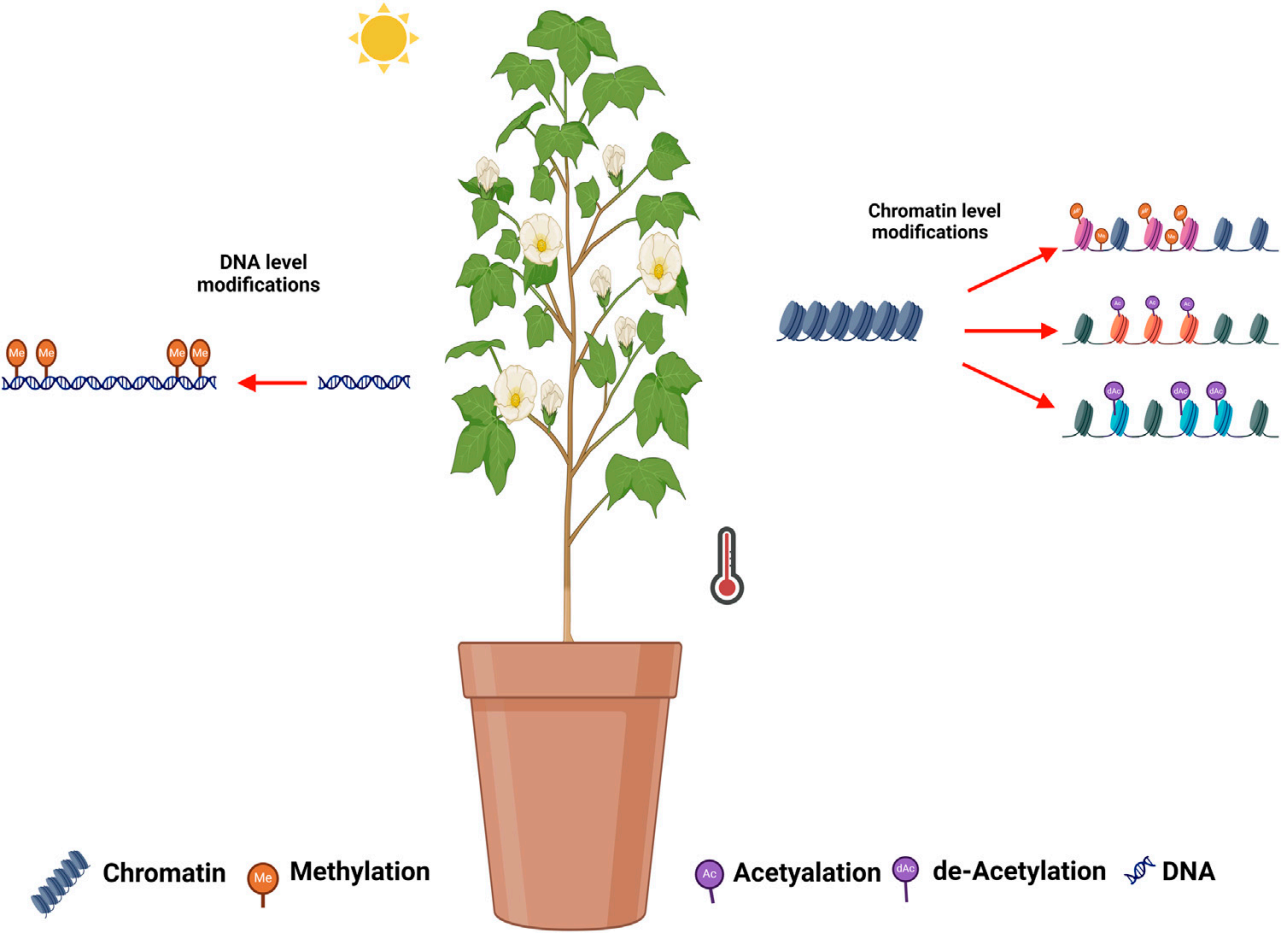
Illustration of epigenetic modifications during HTS in cotton. DNA methylation, histone methylation, histone acetylation, and histone de-acetylation have been reported in cotton in response to HTS in cotton.

In cotton, the floral development stage and high-temperature occurrence coincide, threatening the development of floral organs, i.e., anther and pollen. The epigenetic modifications in response to HTS and the affected genes have been summarized in Table 4 and illustrated in Figure 1. Min et al. (2014) analyzed another development in two cotton genotypes (84,021 and H05) under high and normal temperature (Min et al., 2014). Interestingly, several genes were reported to be associated with DNA methylation and histone modification (acetylation and deacetylation) identified under HTS. Epigenetic modification were found to be involved in anther development, and influenced by high temperature. Ma et al. (2018) observed disruption in DNA methylation (CHH methylation) under HTS conditions in heat sensitive cultivar, which lead to male infertility. Suppression of DNA methylation in sensitive lines under normal temperature also led to male sterility. Disturbing the DNA methylation alters the ROS sugar metabolism and hormonal pathways, which cause abnormalities in pollen development under high HTS. The cotton anther genome displays higher methylation CG, CHG, and CHH sequence. Global methylome shows that promotor-unmethylated genes show higher expression compared to promotor-methylated genes (Ma et al., 2018). Zhang et al. (2020) reported that high temperature induced promoter methylation changes which led to upregulation of the mitochondrial respiratory chain enzyme-associated genes *GhNDUS7, GhCOX6A, GhCX5B2, and GhATPBM*. The increased expression of these genes initiated a sequence of redox processes aimed at generating ATP to support normal anther development under (HTS) (Zhang et al., 2020). Li et al. (2023a) observed widespread disruption in *H3K4me3* and *H3K27me3* modifications in cotton anthers under High-Temperature Stress (HTS), resulting in male sterility. Eliminating *H3K27me3* at the promoters of jasmonate-related genes enhanced their expression, resulting in the restoration of male fertility under HTS in heat-tolerant lines (Li et al., 2023a).

**TABLE 4:** Epigenomic modifications in cotton under HTS.

Specie	Associated gene	Epigenetic marks	Modification	Heat treatment	Tissue	Reference
*G. hirsutum*	*DRM1, DRM3, NERD, NRPD1B, SAHH1, HMT, HAT1, HAT2, HDA15, HDA2C*	DNA Methylation, Histone modification	--	35◦C–39°C: daytime, 29◦C–31°C: night (7 days)	Anther	Min et al. (2014)
*G. raimondii*	*SET domain GrKMTs, GrRBCMTs, GrS-ET*	Histone methylation	H3K9me, H3K4me, H3K36me, H3K27	38 °C	--	Huang et al. (2016)
*G. hirsutum*	*DRM2/CMT2*	DNA Methylation	*CHH-methylation*	39°C–41°C: daytime, 29°C–31°C: night	Anthers	Ma et al. (2018)
*G. hirsutum*	*GhNDUS7, GhCOX6A, GhCX5B2, and GhATPBM*	*DNA- methylation*	*CG, CHG, and CHH- methylation*	36.5 °C: daytime, 28 °C: night	Anther	Zhang et al. (2020)
*G. hirsutum*	*GhAOS, GhAOC2, GhJAZ1*	Histone modification	*H3K27me3, H3K27me3*	38°C–40°C: daytime, 28°C–31°C: night (7 days)	Anther	Li et al. (2023a)

Studies have revealed that epigenetic modifications, including acetylation and methylation, play a substantial role in regulating responses to abiotic stresses and adapting to environmental conditions. The influence of these epigenetic modifications extends significantly to agronomic traits and plant productivity. Unraveling the mechanisms of epigenetic responses in plants has the potential to bring about a significant revolution in crop breeding, especially in the context of climate change (Abdulraheem et al., 2024).

## 7 Phenomics

Rising global temperatures is one of the primary environmental factors that significantly impacts agricultural productivity across the globe. Enhancing our knowledge of the underlying mechanisms that govern how plants respond to heat stress will enable the development of new technologies and breeding approaches to improve plant tolerance to high temperatures (Chen et al., 2022). Previous research has identified several key traits that serve as important indicators of a plant’s tolerance to heat and drought stress (Zhao et al., 2020; Oguz et al., 2022). Evaluating and measuring these tolerance-related traits often requires specialized equipment and advanced technologies. Establishing reliable phenotypic markers of heat stress tolerance that can be evaluated at both the vegetative and reproductive stages would expedite the selection of plant germplasm with enhanced thermotolerance (Gao et al., 2020; Sarkar et al., 2021).

Continuously tracking plant growth and development after heat stress exposure using non-destructive methods like RGB imaging, thermal imaging and chlorophyll fluorescence analysis can offer important insights into physiological responses associated with photosynthetic performance and plant cooling capabilities-insights that would be difficult to gather through visual observation alone. The automated, environmentally-controlled system allows for rapid, efficient screening of large plant populations within a single experiment (Gao et al., 2020). Phenotypic traits, such as plant size, temperature, and photosynthetic efficiency have been successfully applied to evaluate plant performance under drought, salinity and chilling stress (Gao et al., 2020). In a recent study Chen et al. (2019) used high-throughput phenotyping to describe the physiological consequences of heat and drought stress at the early flowering stage of *Brassica rapa*, which identified corbooxylation, phosphate use and flower volume as stress tolerance associated traits (Chen et al., 2019). Hyperspectral imaging of Korean ginseng proved to significantly differentiate the heat tolerant and susceptible and genotypes with maximum precision (Abebe et al., 2023). Arabidopsis hsp101 mutants were exposed to heat stress and phenotype with high-throughput phenotyping system to monitor daily changes in plant morphology and photosynthetic activity in response. Heat stress reduced the quantum yield of PSII and enhanced leaf angle. Longer exposure influenced growth and morphology of plants (Gao et al., 2020).

Therefore, employing image-based phenotyping enables the measurement of diverse traits over time, rendering high-throughput phenotyping appropriate and precise for screening heat stress. Although high-throughput phenotyping has not yet been used to uncover the mechanism by which heat stress affects cotton plants. Cotton breeders have opted to use phenomics coupled with machine learning, which allows automated phenotyping to screen larger cotton populations at early stages and save significant resources compared to conventional phenotyping methods.

Furthermore, conventional phenotyping of cotton has been used only at the reproductive phase to identify heat-tolerant varieties. However, the conventional phenotyping is laborious, time consuming and prone to errors. Thus, the application of HTP and phenomics at earlier stage identification of cotton plants and the ability to monitor daily changes in cotton plants under stress could help identify more tolerant crop varieties in a shorter period of time.

## 8 Combing omics approaches in understanding the response to HTS

**FIGURE 2:**
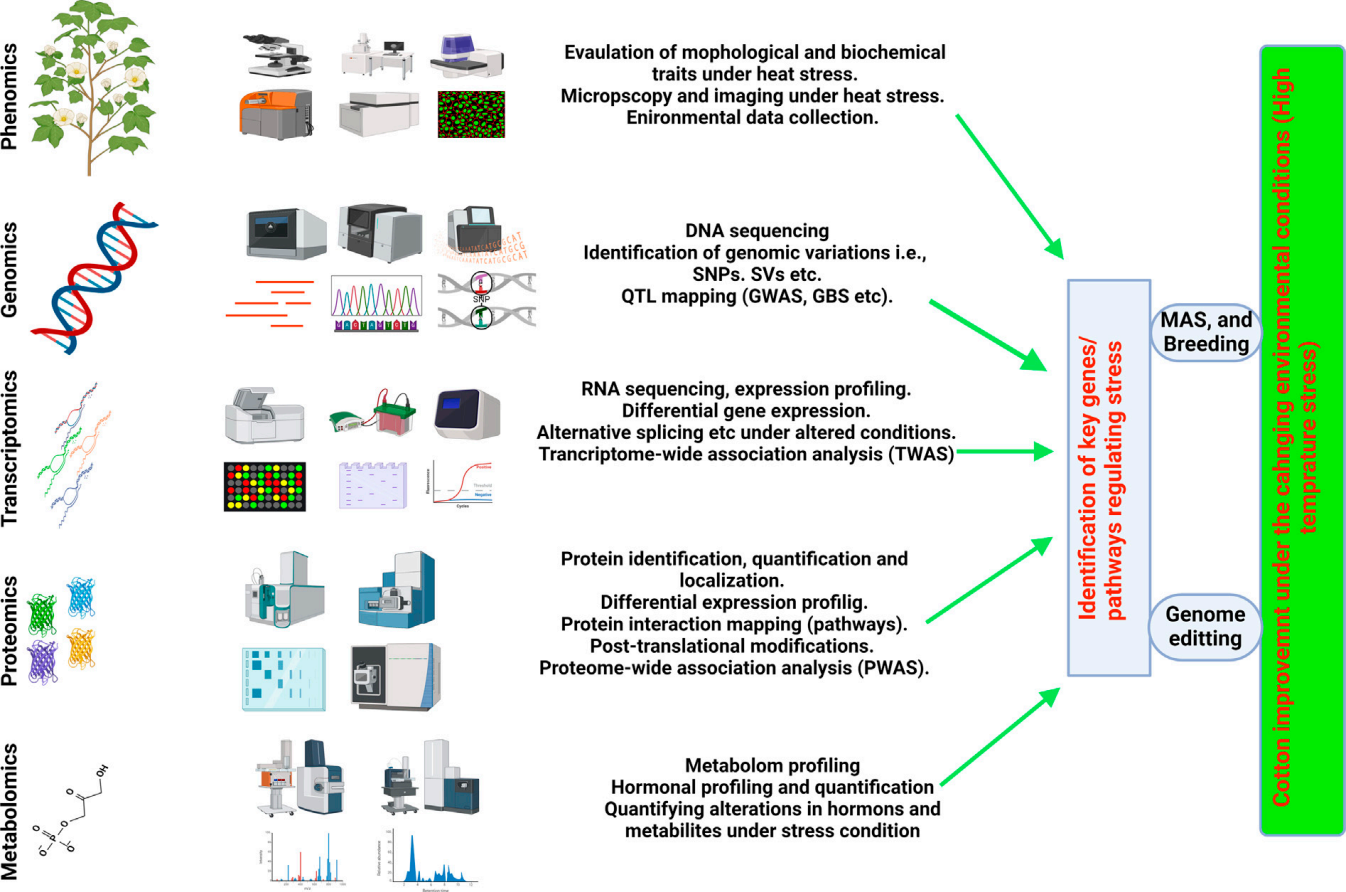
Integrated omics (Multiomic) approaches for understanding HTS response and understanding HTT in cotton and its possible utility in breeding HT-Cotton cultivars.

Omics, accompanied by several modern approaches such as including metabolomics, proteomics, ionomics, and phenomics, are extensively explored in the agricultural sector to gain a deeper understanding of the molecular pathways and cell physiology that govern cell homeostasis and normal functioning (Singh et al., 2021; Yang et al., 2021). Multi-omics techniques, when combined with high-throughput methods, have proven essential for comprehending development, productivity, growth behavior against biotic and abiotic extremes in various crop (Mahmood et al., 2022; Khan et al., 2023). All the techniques mentioned above have been widely used several important crops such as cotton (*Gossypium hirsutum* L.), wheat (*Triticum aestivum* L.), soybeans (*Glycine max*), tomatoes (*Solanum lycopersicum*), barley (*Hordeum vulgare* L.), maize (*Zea mays* L.), and rice (*Oryza sativa* L) (Roychowdhury et al., 2023). The linkage between functional genomics and other omics tools reveals the connections between genomics and phenotypic responses under certain climatic and physiological attributes (Figure 2) (Yang et al., 2021). To assess the lines tolerant to heat stress and having resilience against male sterility induction, the dire need is to evaluate the functionality of anthers and transcriptomes.

The integration of a Genome-Wide Association Study (GWAS) and transcriptome divergence analysis facilitated the identification of three loci associated with heat tolerance. These loci included expression quantitative trait loci (eQTLs) for 13,132 transcripts, 75 protein-coding genes, and 27 long noncoding RNAs. The most effective recognition of 4,820 genes linked to 13 fiber-related variables in cotton by a thorough GWAS has aided in the identification of novel genetic resources for improving fiber quality (Yasir et al., 2019; Li et al., 2021a; Ma et al., 2021b). Concurrently, enriched variants in the gene coding regions and comprehensive expression profiles for genes are provided by parallel transcriptome analysis. These enriched resources of crucial genes prove invaluable in identifying expressions of quantitative trait loci (eQTLs) that may regulate gene expression. The agronomic parameters that play a key role in increasing the growth, biomass, and productivity of cotton were identified by the combined use of GWAS and HRPF approaches (Meena et al., 2022). This reliable method superseded conventional phenomics, offering the sciences of crop genetics and breeding a potent new instrument (Yang et al., 2021) the effectiveness of agronomic features in conjunction with QTL mapping in cotton crop. Concurrently, genetic data and possible phenotyping techniques can offer insights into intricate features to enhance agricultural productivity (Kushanov et al., 2021). At present, limited knowledge is available in cotton on the integration of multiomoc approaches. Limited information of high throughput phenotyping (HTP) and HTP platforms is available in cotton. However, in future the integration of phenomics (high throughput phenotyping) with genomic, transcriptomic, proteomic and other omics will greatly resolve the genetic and molecular and metabolic mechanism of agronomic traits particularly in HTS in cotton. The Figure 2 illustrates how integrated omics (multi-omics) approaches contribute to understanding the heat stress response (HTS) and heat tolerance (HTT) in cotton, as well as their potential utility in breeding HT-Cotton cultivars.

## 9 Future perspective and research on the gap

In the review, we presented the progress in understanding the HTS and its response in cotton. To date, attempts have been made to decipher the genetic, molecular mechanism, and omics, which play a crucial role in understanding the response generated by the cotton plants to HTS. Researchers have utilized genetic (linkage and associating mapping), omics (genomics, transcriptomics, proteomics, and metabolomics), and epigenomic tools for this purpose. Integrative multi-omics has also been employed for this purpose. Together, these provide workable toolkits to develop HTS tolerant cotton cultivars that may cope with the changing environmental conditions and increasing areal temperatures. However, considering the current pace of climate change, growing global population and scarcity of resources, the current progress is lagging behind. Apparently, the pace of research needs to be improved, and progress must be revisited. In light of this review, the following suggestions can be proposed for future studies.

1. High throughput phenotyping and phenomics are gaining attention in crop physiology and breeding. However, the development of HTP platforms and its utility in in cotton is underutilized particularly in stress conditions. There is a dire need to develop HTP platform for growth modeling, non-destructive physiological and morphological assessment of cotton under various growth conditions.
2. Based on the available published information, very limited information is available about the genetics and genetic mechanism of HTS and HTT in cotton. To address this issue, much focus is needed on collecting the germplasm from across the geographical regions and systematically analyzing it under HTS. This will identify tolerant accessions and provide a gene pool for breeding and advance studies. Utilizing this material in genetic and genomics studies will help identify new genes and linked markers related to HTS responses and HTT, which can be used in marker-assisted breeding and gene pyramiding.
3. Transcriptomics and other omics have identified several genes, portions, and metabolites with pivotal roles in generating stress responses, conferring tolerance, and adjusting internal homeostasis under high stress. Several HSP and HSF encoding genes and other DEGs have been identified in cotton in response to HTS, and their pathways have been described. Very few genes have been functionally characterized and utilized in cotton improvement programs.
4. Epigenetic modifications are important in plant development and stress response. In cotton, epigenetic modification has only been studied in floral tissues, particularly in anthers, that cause male sterility. However, the HTS occurrence in cotton at the flowering time has far-reaching impact on yield. Understanding the global epigenomic alteration rather than tissue or stage-specific epigenomic alteration in response to HTS is important. Understanding global epigenomic alteration will enhance our understanding of the HTS and its response in cotton.
5. Integrated omics (or multi-omics) may prove to be more powerful than relying on a single omics tool. Integrated omics studies have been reported to have more power and precision in identifying genes in other crops (maize and rice). Utilizing integrated omics in cotton during HTS may provide a clearer understanding got HTS response, which can be translated into breeding HTT cultivars.
6. Utilization of genome editing technologies for trait engineering may be helpful. CRISPR/Cas-directed evolution (CDE) has been utilized in some crops (rice), which proved effective in generating quantitative tolerance to abiotic stresses. Therefore, identifying genes via omics and further editing with the CDE platform will be an effective and workable strategy for developing climate-resilient cotton cultivars.
